# Longitudinal changes in skeletal muscle in children undergoing cancer treatment: a systematic review and meta-analysis

**DOI:** 10.1007/s00431-025-06349-5

**Published:** 2025-07-31

**Authors:** Anna Maria Markarian, Dennis R. Taaffe, Daniel A. Galvão, Carolyn J. Peddle-McIntyre, Jodie Cochrane Wilkie, Francesco Bettariga, Robert U. Newton

**Affiliations:** 1 Exercise Medicine Research Institute, Edith Cowan University, 270 Joondalup Drive, Joondalup, WA 6027 Australia; 2 School of Medical and Health Sciences, Edith Cowan University, Joondalup, WA Australia; 3 Physical Activity, Sport and Exercise Research Theme, Faculty of Health, Southern Cross University, Gold Coast, Australia; 4 School of Human Movement and Nutrition Sciences, University of Queensland, St. Lucia, QLD 4072 Australia

**Keywords:** Childhood cancer, Skeletal muscle, Body composition, Systematic review and meta-analysis

## Abstract

Skeletal muscle loss during chemotherapy has been associated with poorer outcomes and reduced survival across several types of cancer. However, the extent and progression of muscle loss during treatment for childhood cancers remain unclear. A better understanding could help identify children at increased risk and inform the timing of targeted intervention. This systematic review and meta-analysis aimed to synthesize the evidence on skeletal muscle changes during treatment for childhood cancers and identify factors that influence these outcomes. A systematic search was conducted in CINAHL, Embase, PubMed, SPORTDiscus, and Web of Science. Studies were eligible if they included children and adolescents (< 19 years) undergoing cancer treatment and reported muscle quantity at a minimum of two time points. The methodological quality of the included studies was evaluated using the Newcastle–Ottawa Scale. Twenty studies (*n* = 646; age range: 2.5–14.7 years) were included. A significant decline in muscle quantity was observed during the early phase of treatment (standardized mean difference (SMD): SMD =  − 0.36; 95% CI: − 0.59 to − 0.13; *p* < 0.05). At later follow-up time points, the overall change was not statistically significant (SMD =  − 0.08; 95% CI: − 0.27 to 0.10; *p* = 0.36). However, estimates of muscle quantity varied significantly by assessment modality (*p* = 0.048).

*Conclusion*: Children with cancer experience significant skeletal muscle loss during the intensive phase of treatment. While decrements observed at later time points appear modest, reported outcomes vary considerably depending on the assessment method. Standardized, reliable body composition measures are needed to detect meaningful changes and guide clinical care.

**Table:** 

What is Known: • Skeletal muscle loss during adult cancer treatment is linked to poor outcomes, yet pediatric data are limited and inconsistent.
What is New: • This is the first systematic review and meta-analysis showing significant early-phase skeletal muscle loss in children undergoing cancer treatment, accompanied by concurrent increases in fat mass. • Considerable variability in skeletal muscle estimates across different body composition assessment methods underscores the need for reliable and sensitive measurement techniques to monitor changes accurately and guide clinical care.

**Supplementary Information:**

The online version contains supplementary material available at 10.1007/s00431-025-06349-5.

## Introduction

While advances in therapy and diagnostics over recent decades have significantly improved survival rates for childhood cancer, this improved prognosis may still be compromised by treatment-related complications such as skeletal muscle loss [1–4]. It is well established that skeletal muscle loss during cancer chemotherapy is linked to an increased risk of recurrence and poorer long-term survival in adults [5–8], with emerging evidence suggesting similar risks in pediatric cancer populations [9, 10]. Low skeletal muscle mass is also a risk factor for cardiometabolic conditions in children and adolescents [11–13]. This is particularly concerning for childhood cancer survivors, a population at risk of early onset of age-related health conditions [14] and frailty [15–17]. Consequently, there has been growing interest in targeted interventions, such as exercise medicine, to prevent long-term morbidity in this population [18–20].

Muscle quantity can be described using various terms, including fat-free mass, lean body mass, and skeletal muscle mass; however, these terms are not interchangeable as they refer to different body components [21]. A two-compartment model divides the body into fat and fat-free mass, with the latter encompassing all non-fat tissues, including skeletal muscle, bone tissue, organs, and body water. These models typically rely on densitometric methods (e.g., hydrostatic weighing, air displacement plethysmography) or hydrometric methods (e.g., deuterium dilution, bioelectrical impedance analysis) to estimate body composition based on body density or total body water, respectively [22]. A three-compartment model, such as that used in dual-energy X-ray absorptiometry (DXA), separates the body into fat mass, bone mineral, and bone mineral-free lean body mass [23]. While fat-free mass and lean body mass are often used as proxies for skeletal muscle mass, they include non-muscle tissues and do not exclude inter- and intramuscular fat. In contrast, imaging methods, such as computed tomography (CT) and magnetic resonance imaging (MRI), provide accurate, anatomical, adipose tissue-free measures of skeletal muscle mass from cross-sectional images [21, 23]. Understanding these distinctions is essential, as each term offers a unique perspective on muscle quantity [24].

To date, no comprehensive review has synthesized evidence on longitudinal changes in muscle quantity in children undergoing cancer treatment. A better understanding of the timing and trajectory of muscle loss during treatment could help identify children with impaired muscle development who may benefit from targeted and timely interventions. Therefore, this systematic review and meta-analysis aims to fill this gap by characterizing the direction and magnitude of muscle changes throughout treatment and identifying clinical and methodological factors that influence reported outcomes. Through this synthesis, we aim to guide future research and improve clinical practices supporting muscle health in children undergoing cancer therapy.

## Methods

A meta-analysis was conducted to investigate changes in muscle quantity in children undergoing cancer treatment. All procedures undertaken in this study were reported in accordance with the Cochrane Back Review Group (CBRG) [25] and the Preferred Reporting Items for Systematic Reviews and Meta-Analyses (PRISMA) statement (Online Resource 2) [26, 27].

### Search strategy and study selection procedures

This review was registered in the International Prospective Register of Systematic Reviews (PROSPERO) under the identifier CRD42024551447 as part of a broader protocol for a systematic review and meta-analysis. The protocol aimed to examine the extent of skeletal muscle deficits in (1) children and adolescents undergoing therapy and (2) childhood cancer survivors off cancer treatment. To facilitate a more meaningful synthesis of the evidence, we divided the protocol into two separate reviews: the present systematic review focusing on children undergoing active treatment and a forthcoming meta-analysis on childhood cancer survivors. A systematic search was conducted using CINAHL, Embase, PubMed, SPORTDiscus, and Web of Science databases from inception to June 2024. The search strategy was undertaken with the assistance of a librarian using controlled vocabulary and free-text terms (Online Resource 1). The titles and abstracts were independently evaluated by two authors following the eligibility criteria. Abstracts were selected for full-text evaluation when they did not provide sufficient information. Full-text articles meeting the criteria were retrieved and read independently by both reviewers and were assessed for inclusion in the study. Disagreements regarding the final list of included studies were resolved by consensus.

### Eligibility criteria

We included studies that (a) enrolled children and adolescents < 19 years old diagnosed with any cancer and undergoing cancer treatments; (b) were of prospective, longitudinal design or intervention-based with a usual care control group (for the interventional trials included in the longitudinal analysis only data from the control group were extracted); and (c) reported muscle quantity measures at a minimum of two time points; where studies included multiple follow-up time points, the final available time point was extracted for the primary analysis. Additionally, when studies provided data specifically during the early intensive phase of cancer treatment, typically around 1 to 1.5 months after diagnosis or, in the case of solid tumors, following surgery, this data was also extracted. This approach allowed for the assessment of muscle quantity changes when the most significant losses are expected. No restrictions were placed on the assessment method to allow for an inclusive examination of the literature and the techniques currently used in pediatric oncology. Exclusion criteria included case reports/series, editorials, abstracts, commentaries, and reviews.

### Data extraction

Data extraction was performed by two authors using a structured form in the Covidence systematic review platform [28]. The review protocol was embedded in Covidence to ensure that study eligibility was consistently compared against the predefined criteria. Inconsistencies in data extraction were resolved during weekly meetings. Study information, including sample size, study design, type of cancer diagnosis, age at cancer diagnosis, height, and weight, was extracted along with the outcomes of interest. While the primary outcome was muscle quantity, we also took the opportunity to examine changes in fat and total body mass based on the available data from the included studies. Data were extracted as mean and standard deviation (SD) or in a manner that allowed transformation into mean and SD. In studies where medians, ranges, or interquartile ranges were provided instead of the mean and SD, these were estimated using the formula from Wan et al. [29]. When the standard error (SE) was reported instead of SD, the latter was obtained using the formula from Altman and Bland [30]. For studies that followed a normal distribution and provided the mean and confidence interval (CI), we transformed the confidence interval into SE and then calculated the SD according to Cochrane recommendations [31]. Finally, when graphs were presented instead of numerical data, data were extracted from the plots using a specific tool for data extraction (WebPlotDigitizer, San Francisco, CA) [32].

### Quality assessment

The methodological quality of the included studies was independently assessed by two authors using the Newcastle–Ottawa Scale (NOS) [33]. Scores range from 0 to 9, with higher scores indicating higher quality (0–3 points low quality, 4–6 points moderate quality, 7–9 points high quality).

### Statistical analysis

A three-level mixed-effects meta-analysis was conducted, with individual studies included as random effects. Cluster robust point estimates and 95% CIs are reported and weighted by inverse sampling variance to account for the within- and between-study variance (tau^2^). Additionally, a restricted maximal-likelihood estimation was used in all models. Statistical significance was assumed when the standardized mean difference (SMD) was below an alpha level of *p* < 0.05. Statistical heterogeneity was assessed using the Cochran *Q* test, with an *I*^2^ greater than 50% indicating high heterogeneity. Publication bias was explored using contour-enhanced funnel plots and Egger’s test [34]. Heterogeneity, publication bias, sensitivity, and moderator analyses were performed to substantiate the results. Subgroup analyses were conducted for (a) assessment method and (b) measure of muscle quantity. Multilevel models with robust estimates were generated for each subgroup, and fixed effects with the moderator’s model were used for comparison. Analyses were conducted using the meta [35], metafor [36], and clubSandwich [37] packages in R (R Core Team, version 4.3.3., 2024).

## Results

**Fig. 1:**
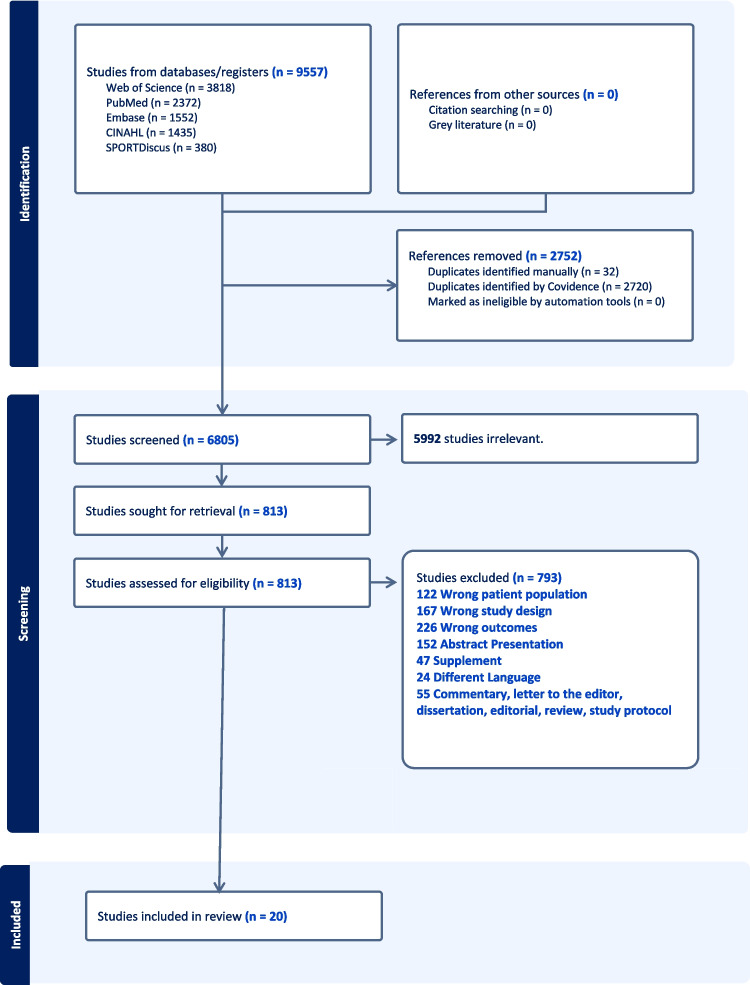
Flow chart of study selection process

A total of 9557 studies were retrieved from our search. After removing duplicates, 6805 records remained for title and abstract screening. Of these, 5992 were excluded because they were irrelevant to our research question. An additional 793 records were excluded for specific reasons, as detailed in Fig. 1. As a result, 20 studies were included in this review [38–57].

### Study characteristics

A general description of the characteristics of the 20 studies (646 participants) is provided in Table 1. The articles were published between 1990 and 2024, and participants’ ages ranged from 2.5 to 14.7 years. The techniques used to estimate skeletal muscle quantity in children with cancer included CT (*n* = 7), DXA (*n* = 6), bioelectrical impedance analysis (*n* = 3), deuterium dilution (*n* = 2), ultrasound (*n* = 2), and skinfolds (*n* = 2). NOS scores are presented in Online Resource 3.

**Table 1:** Characteristics of the studies examining muscle quantity in children undergoing cancer treatment

Author, year, country	Study design	Participant characteristics (***n***, sex, cancer type, age in years, height in cm, weight in kg)	Assessment technique	Time interval(s) included in this review
Koskelo et al., 1990, Finland [56]	Prospective cohort with cross-sectional healthy controls	14 (8 males) children newly diagnosed with acute leukemia; Mean age at study: 5.9; Height: NG; Weight: NG	Muscle Index (cm^2^/m^2^) from US	6 weeks (early treatment) and 24 weeks post diagnosis
Taskinen et al., 1998, Finland [54]	Prospective cohort with cross-sectional healthy controls	19 (males NG) children newly diagnosed with solid tumours; Mean age at study: 6.2; Height: NG; Weight: NG	Muscle Index (cm^2^/m^2^) from US	1–2 months after operation
Barbosa-Cortés et al., 2007, Mexico [40]	Prospective cohort	8 (5 males) children with lymphoma and 9 (4 males) children with solid tumours; Mean age at study: 11.1 (Lym), 9.5 (ST); Height: 140.2 (Lym), 136.9 (ST); Weight: 33.2 (Lym), 32.3 (ST)	Fat-free and fat mass (kg) from deuterium dilution	~ 6 months after the first chemotherapy course (193 ± 12 days for lymphoma; 187 ± 22 days for solid tumors)
Bourgeois et al., 2008, Canada [42]	Retrospective cohort (natural history group)	50 (35 males) children with childhood acute lymphoblastic leukemia during maintenance; Mean age at study: 5.9; Height: 117.7; Weight: 24.1	Fat-free mass (kg) from DXA	38-week interval within maintenance phase
Zaid et al., 2012, Malaysia [38]	Randomized controlled trial	25 (13 males) children with leukemia; Mean age at study: NG; Height: 122; Weight: 21.7	Mid-upper arm circumference (cm) from skinfold	60-day interval (phase unspecified)
Fuemmeler et al., 2013, USA [43]	Prospective cohort with longitudinal follow-up of both cases and controls	15 (8 males) children with newly diagnosed lymphoma and leukemia; Mean age at study: 10.3; Height *Z*-score: 0.4; Weight *Z*-score: 0.57	Lean body mass and fat mass (kg) from DXA	12 months post diagnosis
Orgel et al., 2018, USA [45]	Prospective cohort	50 (30 males) children newly diagnosed with leukemia; Mean age at study: 14.7; Height: NG; Weight: 45.4	Lean body mass and fat mass (kg) from DXA	Diagnosis to end of induction (early treatment), and to end of delayed intensification (intervals not specified)
Waked et al., 2018, KSA [47]	Randomized controlled trial	23 (18 males) children with acute lymphoblastic leukemia; Mean age at study: 9.9; Height: 129.3; Weight: 32.3	Lean body mass (kg) from DXA	12-month interval within maintenance phase
Suzuki et al., 2018, Japan [53]	Retrospective cohort	47 (24 males) children with acute lymphoblastic leukemia; Mean age at study: 8.5; Height: 129.3; Weight: 35.8	Psoas major area (mm^2^) from CT	Pre- to post-induction therapy (35 days)
Mueske et al., 2019, USA [49]	Prospective cohort with cross-sectional healthy controls	12 (5 males) children newly diagnosed with acute lymphoblastic leukemia; Mean age at study: 14.4; Height: 158.8; Weight: 52.4	Muscle and total adipose volume (cm^3^) from CT	Diagnosis to end of induction (early treatment, ~ 28–35 days), and to end of delayed intensification (~ 7–9 months)
Yang et al., 2019, Korea [46]	Prospective cohort with cross-sectional healthy controls	19 (14 males) children newly diagnosed with hematologic malignancies and 11 (7 males) survivors of solid tumours; Mean age at study: 10.8 (HM), 11.6 (ST); Height: 137.9 (HM), 154.5 (ST); Weight: 36.6 (HM), 42.2 (ST)	Lean body mass and fat mass (kg) from DXA	1 and 12 months post diagnosis
Behling et al., 2020, Brazil [41]	Prospective cohort	14 (7 males) children with hematologic and solid tumours; Mean age at study: 10.9 (HM), 9.1 (ST); Height: 145 (HM), 132 (ST); Weight: 34.05 (HM), 27.5 (ST)	Fat-free and fat mass (kg) from deuterium dilution and bioelectrical impedance; mid-upper arm muscle circumference (cm)	6-month interval (phase unspecified)
IJpma et al., 2021, The Netherlands [51]	Retrospective cohort	29 (19 males) children newly diagnosed with neuroblastoma; Mean age at study: 3.2; height-for-age (SDS), median (IQR): − 0.3 (− 0.8 to − 0.5); Weight-for-age (SDS), median (IQR): − 0.5 (− 1.4 to 0.3)	Muscle CSA (cm^2^) from CT	1 and 13 months post diagnosis*
Nakamura et al., 2021, Japan [55]	Retrospective cohort	24 (16 males) children newly diagnosed with neuroblastoma; Mean age at study: 2.5; Height: NG; Weight: NG	Psoas major index (cm^2^/m^2^) from CT	Diagnosis and after first chemotherapy cycle
Saultier et al., 2021, France [48]	Randomized controlled trial	39 (23 males) children with childhood cancer (mixed diagnoses); Mean age at study: 11.2; Height: NG; Weight: NG	Lean and fat mass (kg) from impedance	6 months after T0 (T0 ~ 9.3 months post diagnosis)
Romano et al., 2022, Italy [52]	Retrospective cohort	21 (11 males) children with bone and soft tissue sarcoma; Mean age at study: 10.7; Height: NG; Weight: NG	Psoas major area (mm^2^) from CT	12 months post diagnosis
Wadhwa et al., 2023, USA [50]	Retrospective cohort	78 (49 males) children with lymphoma and rhabdomyosarcoma; Mean age at study: 12.3 Height: NG; Weight: NG	Skeletal muscle index (cm^2^/m^2^) and height adjusted adipose tissue (cm^2^/m^2^) from CT	Pre-chemotherapy and 48 days post (median time between scans)
Kellerman et al., 2023, South Africa [44]	Prospective cohort	43 (22 males) children with childhood cancer (mixed diagnoses); Mean age at study: 5.4; Height: 103.8; Weight: 16.6	Fat-free and fat mass (kg) from bioelectrical impedance	1 and 5 months post diagnosis
Barbosa-Cortés et al., 2023, Mexico [57]	Randomized controlled trial	22 (10 males) children newly diagnosed with acute lymphoblastic leukemia; Mean age at study: 7 years; Height: 121.7; Weight: 29.5	Lean body mass (kg) from DXA	~ 1.5 months (day 44) and ~ 3 months (day 91) post diagnosis
Bang et al., 2024, South Korea [39]	Retrospective cohort	70 (45 males) children with newly diagnosed HR neuroblastoma; Mean age at study: 3.8; Height: NG; Weight percentile: 45.04	Psoas major area (mm^2^) from CT	Two timepoints ~ 13 months apart (12.9 ± 1.7 months post diagnosis)

*NG*, not given; *ST*, solid tumour; *Lym*, lymphomas; *HM*, hematologic malignancy; *ALL*;, acute lymphoblastic leukemia; *SDS*, standard deviation score; *IQR*, interquartile range; *CT*, computed tomography; *US*, ultrasound; *DXA*, dual- energy X-ray absorptiometry.

Notes: Weight (total body mass) was measured using electronic (also referred to as digital) scales

Sample sizes may vary between total cohort size and the number of participants for specific measurement; Where original studies reported median age, height, and weight values, these have been converted to estimated means using the Wan et al. [29] formula for consistency

*Excluded from meta-analysis due to reporting format (median and confidence intervals only)

### Changes in skeletal muscle mass

**Fig. 2:**
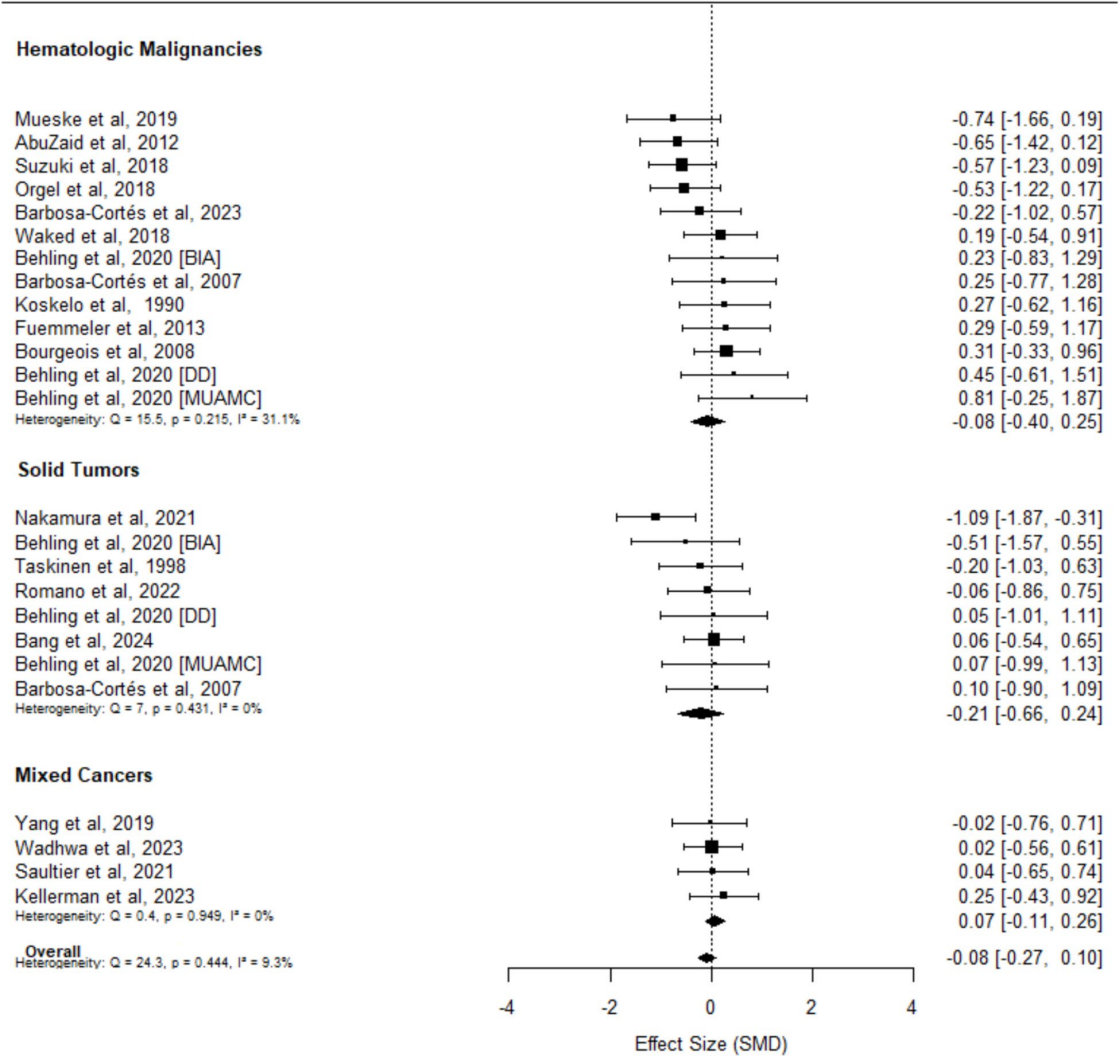
Forest plots of changes in muscle quantity among children undergoing cancer treatment

The overall pooled SMD was − 0.08 (95% CI − 0.27 to 0.10; *p* = 0.36; Fig. 2) based on 25 effect sizes across 19 studies. Heterogeneity (*I*^2^) was 9.30%, and the contour-enhanced funnel plot did not indicate the presence of publication bias (*τ* = 0.20; *p* = 0.89; Online Resource 4). Within diagnostic subgroups (Fig. 2), the pooled SMD was − 0.08 (95% CI − 0.40 to 0.25; *p* = 0.61; *I*^2^: 31.10%) for hematologic cancers, − 0.21 (95% CI − 0.66 to 0.24; *p* = 0.27; *I*^2^: 0%) for solid tumors, and 0.07 (95% CI − 0.11 to 0.26; *p* = 0.30; *I*^2^: 0%) for mixed cancer diagnoses.

**Fig. 3:**
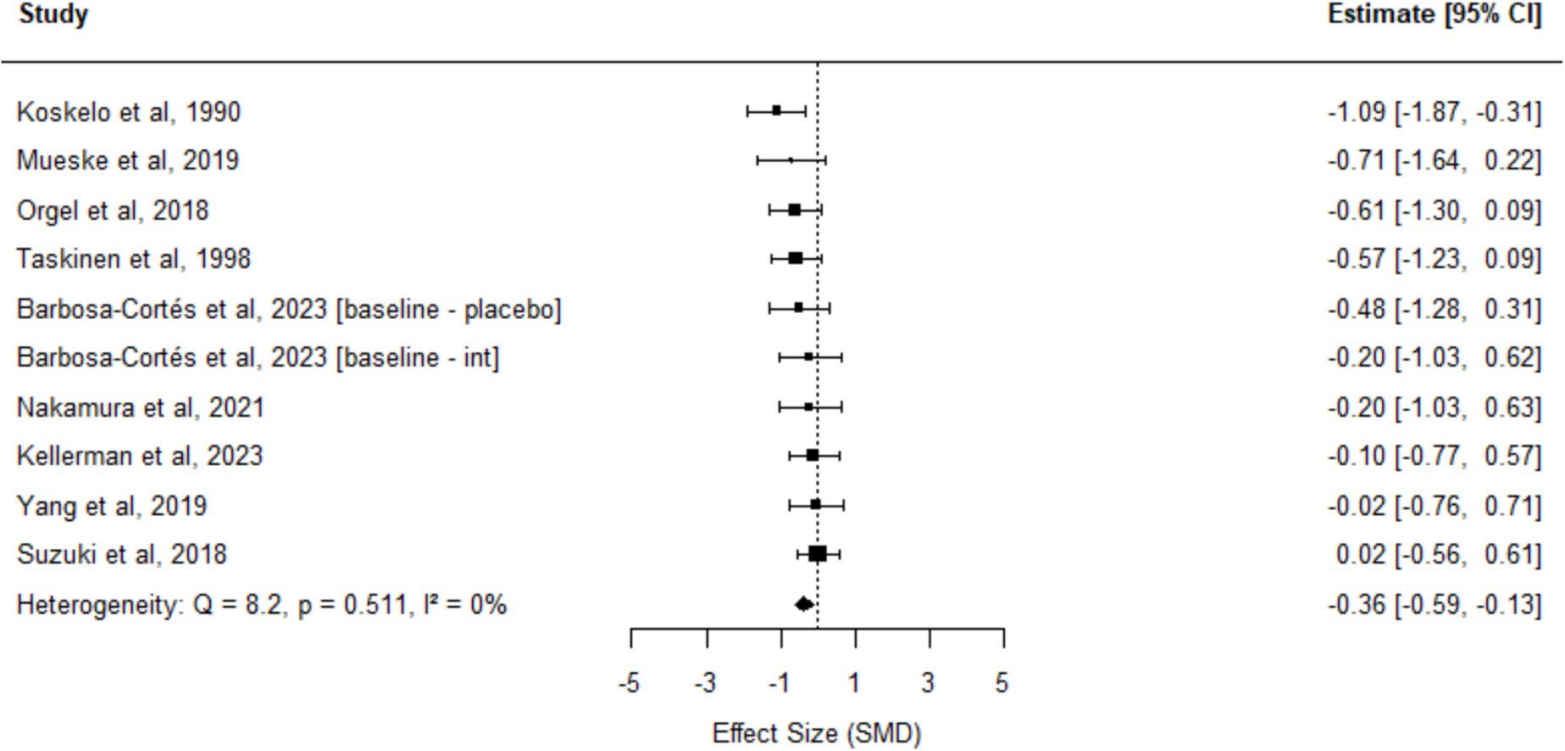
Forest plot of changes in muscle quantity during the initial treatment phase in children diagnosed with cancer

In contrast, analysis of the early, intensive phase of treatment (< 1.5 months after diagnosis) revealed a significant decline in muscle quantity, based on 10 effect sizes across 9 studies (SMD =  − 0.36; 95% CI: − 0.59 to − 0.13; *I*^2^: 0%; Fig. 3), confirming the greater muscle loss early during cancer therapy.

Cancer type was not a significant moderator of muscle quantity estimates (*p* = 0.21). In contrast, assessment modality showed a significant moderating effect (*p* = 0.048), whereas the anatomical scope of the measure (regional vs. total body) did not (*p* = 0.09). This suggests that the measurement method, rather than the body region assessed, influences reported muscle quantity outcomes. The overall and subgroup analyses and results of tests of moderators are presented in Online Resource 5 and 6.

### Changes in total body and fat mass:

**Fig. 4:**
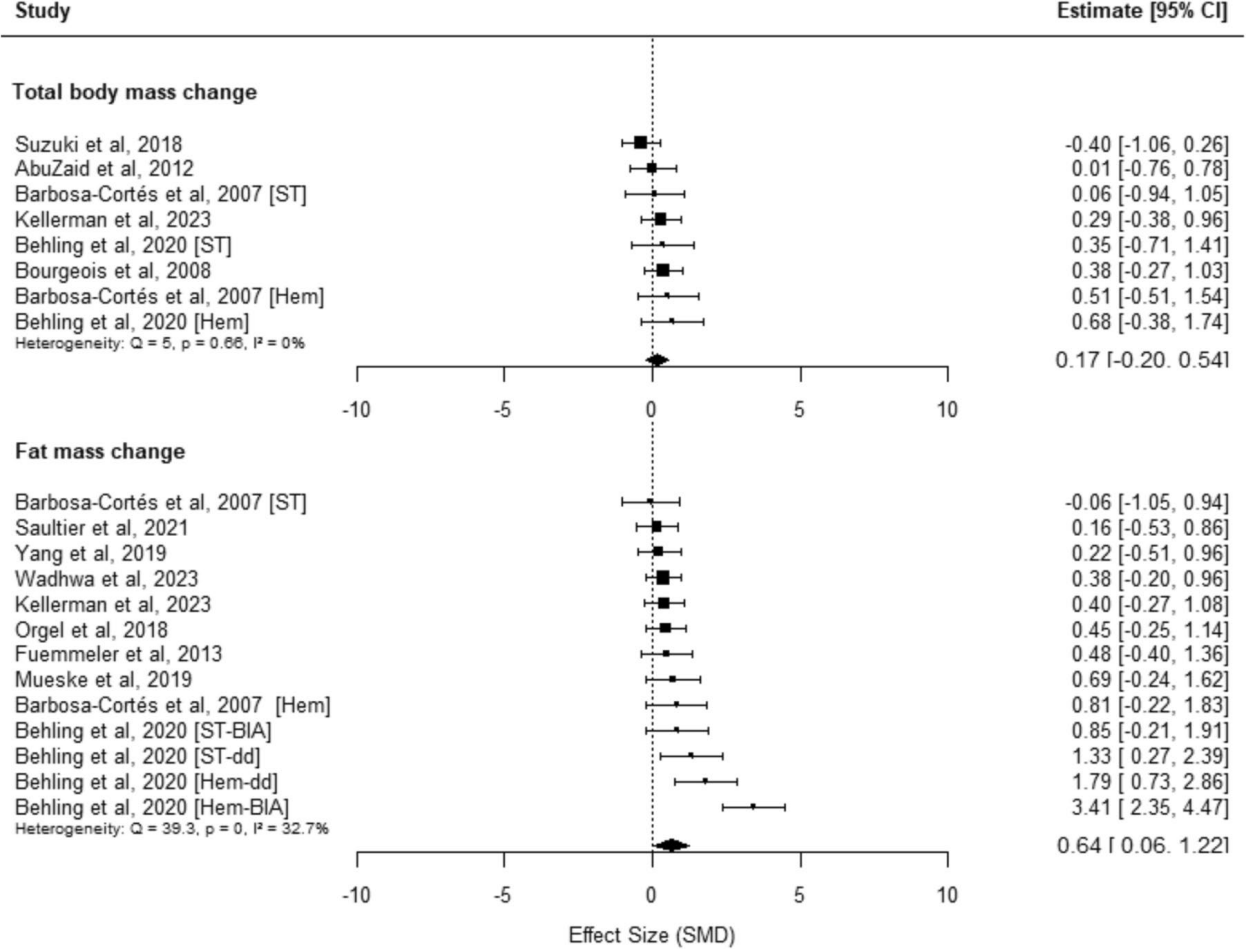
Forest plots of changes in total body mass and fat mass among children undergoing cancer treatment

The overall pooled SMD for total body mass change was 0.17 (95% CI: − 0.20 to 0.54; *p* = 0.28; *I*^2^: 0%; Fig. 4) based on 8 effect sizes across 6 studies. In contrast, the pooled SMD for fat mass change was 0.64 (95% CI: 0.06 to 1.22; *p* = 0.03; *I*^2^: 32.7%; Fig. 4) based on 13 effect sizes from 9 studies, indicating a significant increase in fat mass despite only minimal increases in total body mass.

## Discussion

This systematic review and meta-analysis provides the first comprehensive overview of changes in muscle quantity among children undergoing cancer treatment. Our study has three key findings. First, children with cancer experience significant declines in muscle quantity during the early phase of cancer therapy. Second, while muscle quantity appears to improve at later stages of cancer treatment, this recovery is often accompanied by marked increases in fat mass. Third, muscle quantity estimates vary systematically across body composition assessment methods, underscoring the need for standardized, reliable, and sensitive measurement techniques to monitor changes accurately and guide clinical care.

These findings have important clinical implications, as secondary sarcopenia, i.e., muscle loss associated with malignant disease, is a contributor to increased mortality, surgical complications, and heightened chemotherapy toxicity [58]. The risk of drug-related toxicity can increase, particularly because chemotherapy dosing is primarily based on body surface area (derived from weight and height) and does not account for underlying body composition [59]. The studies within this review consistently demonstrated that muscle loss during the initial phases of cancer treatment is accompanied by increases in fat mass [44–46, 49, 56, 57]. Such shifts in body composition can create a mismatch between dosing assumptions and actual metabolic capacity, heightening the risk of treatment-related complications and poorer clinical outcomes in patients with diminished skeletal muscle mass.

While we found no significant changes in skeletal muscle between initial and later time points during treatment, caution is warranted when interpreting these findings, as the absence of a normative reference or healthy comparison group may obscure clinically meaningful deficits. In studies that included a healthy control group or provided *Z*-scores alongside absolute measures, children with cancer consistently began treatment with significantly lower muscle mass than their healthy peers [39, 43, 46, 49, 52]. As such, apparent recoveries may be clinically misleading as they would not reflect healthy muscle development. This interpretation is supported by the literature showing that childhood cancer survivors experience persistent deficits in muscle strength and balance [60], altered body composition [61], and reduced fitness and physical activity participation compared to their peers [62–64].

Another critical consideration when investigating recovery trends is that improvement in muscle quantity alone does not necessarily indicate healthy growth, particularly when accompanied by a disproportionate increase in adiposity. Overweight and obese individuals typically exhibit higher absolute muscle mass than normal-weight individuals, given that weight gain comprises muscle and fat. However, across various stages of cancer treatment, children with cancer exhibited concurrent increases in body fat, regardless of whether muscle quantity improved [40, 41, 43, 44], declined [45, 46, 49], or remained stable [48, 50, 51]. The direction of effects for fat mass was broadly consistent across studies, and our pooled within-group data confirmed significant increases in fat mass despite only modest gains in total body mass. These findings indicate a potential disadvantage in body composition for children with cancer in terms of relative muscle proportion (% lean body mass) and overall adiposity. Such patterns could signal early signs of metabolic dysregulation rather than healthy developmental progression.

The dissociation between fat and muscle during cancer treatment may also manifest through changes in muscle quality. Although data remain limited, preliminary findings suggest increases in muscle-associated adipose tissue [49] and a decrease in CT-derived skeletal muscle density [50] during cancer treatment, indicating declines in muscle integrity beyond measurable changes in muscle quantity. Imaging techniques such as MRI and CT offer valuable insights into muscle density and inter- or intramuscular adipose tissue areas, providing information on metabolic health. However, their application in longitudinal research is often constrained by cost, limited availability, and the need for specialized personnel for image acquisition and analysis. CT also involves ionizing radiation, which restricts its use for repeated measurements.

As a more accessible alternative, peripheral quantitative computed tomography (pQCT) has recently gained attention for evaluating soft tissue composition [65, 66]. It can quantify muscle density, subcutaneous fat, and intermuscular adipose tissue while exposing participants to significantly lower radiation doses compared to CT [67]. Blew et al. [68] demonstrated strong correlations between pQCT-derived measurements of thigh muscle density and intermuscular adipose tissue and corresponding MRI-derived values in young girls. However, correlations for intramuscular adipose tissue were weaker, possibly due to the lower resolution of pQCT than MRI. Future research may explore the utility of pQCT in assessing muscle quality and composition in children undergoing cancer treatment and consider evaluating the reliability of intramuscular adipose tissue at the tibia, where a smaller cross-sectional area may improve resolution and lead to more reliable estimates.

Our subgroup analysis further supports that imaging methods, such as CT, may offer more accurate and sensitive assessments of temporal muscle changes during cancer treatment. This was evident in our analysis, which showed that reductions in skeletal muscle were more pronounced when CT was used. CT offers anatomical, adipose tissue-free measurements of muscle cross-sectional area or volume. In contrast, hydration-sensitive methods, such as DXA, may be influenced by fat infiltration and shifts in fluid balance, leading to inaccuracies in muscle estimates. These limitations extend to fat assessment, where CT offers precise volumetric measurements of *adipose tissue* compartments, whereas DXA yields broader *fat* estimates [69–72]. Together, these methodological differences underscore the importance of interpreting changes in body composition within the context of the measurement technique used.

Importantly, interpreting serial body composition measurements depends on the knowledge of the smallest change beyond the threshold for error [73]. Without incorporating the least significant change values, it becomes difficult to determine whether observed changes reflect physiological shifts or merely fall within the bounds of technical variability inherent to the measurement technique. This complicates the interpretation of statistically significant changes that may not be clinically meaningful or, conversely, that may be clinically meaningful but do not reach statistical significance due to increased variability (e.g., population heterogeneity). As such, future studies should prioritize reporting the least significant change values and reliability metrics (e.g., intra- and inter-rater reliability, coefficient of variation) for all body composition assessment methods.

Accurate interpretation of body composition is essential to guide and implement appropriate countermeasures, such as exercise therapies. Without recognizing the extent and nature of body composition alterations, interventions to address these concerns may not be prioritized or implemented. To date, preventive strategies remain underutilized in children undergoing cancer treatment, despite evidence supporting their safety and effectiveness [74–78]. A stronger focus on identifying which children are most predisposed to muscle loss and most likely to benefit from early intervention will be critical to realizing the full potential of these approaches.

### Strengths, limitations, and risk of bias

This is the first systematic review to synthesize prospective longitudinal studies evaluating temporal changes in muscle quantity among children undergoing cancer treatment. The topic is particularly relevant given the established associations between muscle loss and poorer functional outcomes, metabolic complications, and reduced quality of life in childhood cancer survivors. Nonetheless, several limitations are worthy of comment. First, this meta-analysis focused on within-group changes rather than between-group comparisons (i.e., children with cancer vs. healthy controls), as few studies included a healthy comparator group. However, it is essential to recognize that between-group comparisons can also be limited, particularly by baseline differences in skeletal muscle between children with cancer and their healthy peers. Thus, caution is warranted when interpreting longitudinal changes, regardless of the analytic approach. Second, our analysis was conducted at the study rather than the individual patient level. This limits the ability to account for variability in factors such as treatment protocols, age, and hormonal status, all of which influence muscle development. For instance, most studies include broad age ranges, spanning early childhood to adolescence, and report only group mean ages despite considerable variation in developmental stages within these ranges. However, it is also worth noting that cancer treatments may delay the onset or progression of puberty, making comparisons based solely on age- and sex-matched norms potentially misleading. Incorporating pubertal staging in future research would allow a more accurate interpretation of developmental deviations. Lastly, there was considerable variability in the body composition assessment techniques and protocols across studies, with few reporting on the reliability or standardization of their measurement procedures. Notably, our subgroup analysis revealed that different assessment methods vary in sensitivity to detect temporal changes in muscle quantity. Thus, we highlight an important methodological consideration and suggest that future studies further investigate how the measurement approach influences the interpretation of muscle alterations during cancer treatment.

## Conclusion

This systematic review and meta-analysis highlights significant muscle loss during the early phase of childhood cancer treatment, accompanied by concurrent increases in fat mass. Although muscle quantity appeared to recover to baseline levels later in treatment, subgroup analyses revealed considerable variability in muscle estimates across different body composition assessment methods. These findings underscore the need for further validation of body composition tools in pediatric oncology to improve the early detection of muscle deficits and support targeted interventions aimed at preserving muscle health throughout treatment and into survivorship.

## Supplementary Information

Below is the link to the electronic supplementary material.

ESM 1 (DOCX 343 KB)

## Data Availability

Data availability statement: The data that support the findings of this study are available from the corresponding author upon request.
